# Fostering the delivery of non‐pharmacological nursing home dementia care: Designing and executing a multi‐site pragmatic clinical trial

**DOI:** 10.1002/alz.083709

**Published:** 2025-01-09

**Authors:** Natalie E Leland, Victoria Shier, Cara A. Lekovitch, Yuna H. Bae‐Shaaw, Neeraj Sood, Claire E Day, Paul Cass, Dominique H. Como, Carin M. Wong, Felicia M Chew

**Affiliations:** ^1^ University of Pittsburgh, Pittsburgh, PA USA; ^2^ University of Southern California, Los Angeles, CA USA; ^3^ Alzheimer’s Association, San Jose, CA USA; ^4^ Genesis, Kennett Square, PA USA; ^5^ Thomas Jefferson University, Philadelphia, PA USA

## Abstract

**Background:**

Nursing home (NH) staff face daily challenges caring for the residents living with dementia (e.g., management of behavioral and psychological symptoms). Research supports the delivery of *team‐* and *problem‐based* non‐pharmacological approaches to dementia care. The team‐based approach includes dementia training for all NH staff using a common language and strategies to support continuity and sustainability. The problem‐based approach capitalizes on the discipline‐specific expertise of the professional healthcare providers (e.g., rehabilitation) to target emergent issues. Prior work has not compared the two approaches. Our objective is to determine if there is a difference between these non‐pharmacological dementia care approaches with respect to resident outcomes and satisfaction and acceptability among NH staff and family caregivers.

**Method:**

We will describe the development and execution of a community‐engaged pragmatic clinical trial that compared team‐ and problem‐based approaches to NH dementia care. The trial leveraged a convergent mixed methods design in order to examine (a) comparative effectiveness of two dementia care approaches on long‐term NH resident outcomes (e.g., off‐label antipsychotic medications), (b) whether either approach was protective against the negative consequences of COVID‐19, and (c) NH staff and family caregiver perspectives of the care approaches (e.g., application, recommendations, sustainability). Data collection included the NH Minimum Data Set, electronic medical records, and interviews with NH staff and families of residents living with dementia.

**Result:**

80 NHs were randomized to one of the two treatment approaches. This presentation will share considerations for designing and implementing pragmatic trials in a United States NH context. Designing this NH pragmatic trial required a multi‐level implementation framework, which accounted for variations in societal, contextual, and encounter level factors related to the approaches as well as peripheral factors impacting NH care more broadly. Successful completion of the study required the active engagement of a community‐engaged Advisory Committee.

**Conclusion:**

The design and execution of NH implementation research should consider intervention‐specific and peripheral factors spanning levels of influence and be anchored in community‐engagement methodology. Results of the trial can provide health system leaders and policymakers with evidence on how to advance dementia care in an effort to optimize resident outcomes.

